# The European experience with testing and surveillance during the first phase of the COVID-19 pandemic

**DOI:** 10.1186/s12992-023-00950-9

**Published:** 2023-07-21

**Authors:** Michael A. Stoto, Chiara Reno, Svetla Tsolova, Maria Pia Fantini

**Affiliations:** 1 Department of Health Management and Policy, Georgetown University, Washington, D.C United States of America; 2 Harvard T.H. Chan School of Public Health, Boston, United States; 3 Department of Biomedical and Neuromotor Sciences, Alma Mater Studiorum, Università di Bologna, Bologna, 40126 Italy; 4 Interdisciplinary Research Center “Health Science”, Scuola Superiore Sant’Anna, Pisa, Italy; 5 European Centre for Disease prevention and Control (ECDC), Solna, Sweden

**Keywords:** Preparedness, Resilience, Testing, Contact tracing, Surveillance, ECDC, COVID-19, Pandemic

## Abstract

**Background:**

COVID-19 pandemic provides a unique opportunity to learn the challenges encountered by public health emergency preparedness systems, both in terms of problems encountered and adaptations during and after the first wave, as well as successful responses to them.

**Results:**

This work draws on published literature, interviews with countries and institutional documents as part of a European Centre for Disease Prevention and Control project that aims to identify the implications for preparedness measurement derived from COVID-19 pandemic experience in order to advance future preparedness efforts in European Union member states. The analysis focused on testing and surveillance themes and five countries were considered, namely Italy, Germany, Finland, Spain and Croatia. Our analysis shown that a country’s ability to conduct testing at scale was critical, especially early in the pandemic, and the inability to scale up testing operations created critical issues for public health operations such as contact tracing. Countries were required to develop new strategies, approaches, and policies under pressure and to review and revise them as the pandemic evolved, also considering that public health systems operate at the national, regional, and local level with respect to testing, contact tracing, and surveillance, and involve both government agencies as well as private organizations. Therefore, communication among multiple public and private entities at all levels and coordination of the testing and surveillance activities was critical.

**Conclusion:**

With regard to testing and surveillance, three capabilities that were essential to the COVID-19 response in the first phase, and presumably in other public health emergencies: the ability to scale-up testing, contact tracing, surveillance efforts; flexibility to develop new strategies, approaches, and policies under pressure and to review and revise them as the pandemic evolved; and the ability to coordinate and communicate in complex public health systems that operate at the national, regional, and local level with respect and involve multiple government agencies as well as private organizations.

## Background

With more than 600 million people infected and 6 million deaths worldwide (as of October 2022), as well as disrupted economies, lost schooling, and many other effects, it is hard to think of Coronavirus disease 2019 (COVID-19) as anything but a disaster [1]. The pandemic, however, does present a rare opportunity to observe a full-blown public health emergency response, and to identify the preparedness capacities and capabilities of European Union (EU) Member States in action.

Effectively responding to emerging health threats requires a strong national response as well as effective coordination across countries. In 2013, the European Union (EU) adopted Decision No. 1082/2013/EU (Decision 1082), which seeks to strengthen public health emergency preparedness and response planning within and across EU Member States. Currently, in light of challenges experienced in the COVID-19 crisis, the EU is adopting legislation to strengthen both Union’ and the Member States collective ability to respond to future communicable disease threats [2].

This report describes part of a project undertaken on behalf of the European Centre for Disease Prevention and Control (ECDC). The goal of this analysis is to identify specific challenges that were experienced by five European countries, as well as successful responses to them, and to identify the implications for preparedness measurement in order to advance future preparedness efforts in the EU Member States. The goal is to capitalize on the experience of countries to identify specific preparedness capacities and capabilities that were essential in the response, and thus should be included in preparedness indicators. It is not to assess or judge any specific Member State’s response.

The ECDC project focused on four areas and this report summarizes the findings with respect to two: testing and surveillance. Parallel reports address the other two areas of healthcare sector coordination and emergency risk communication [3]. The implications of these results for preparedness measurement are discussed in an ECDC technical report [4].

## Methods

Assessing preparedness is challenging because serious public health emergencies and cross-border event are relatively infrequent and possess many aspects specific to their respective contexts, leaving few opportunities to assess outcomes by direct observation in after action reviews (AARs). Consequently, statistical approaches common in other areas of healthcare (e.g. post-surgical mortality or proportions of patients receiving an indicated preventative service) are not available [5]. Moreover, PH systems are multi-jurisdictional and multidisciplinary and vary markedly from one EU country to another. Public health agencies operate at the national, regional, and local levels, and require multi-national collaboration in cases of cross-border outbreaks. Other entities that support public health emergency preparedness include key players in the health sector (e.g. hospitals and primary care physicians) as well as counterparts in non-health sectors. Consequently, responsibility and accountability for public health emergency preparedness is diffuse, making it difficult to determine which partner’s performance to measure, and how to hold each partnering entity accountable for its contributions [6].

In order to address these measurement challenges, we used the ECDC Preparedness Logic Model [7], which distinguishes between preparedness response capabilities and capacities. *Capabilities* describe the actions a public health system is capable of taking to effectively identify, characterize, and respond to emergencies such as providing testing for diagnostic and epidemiological purposes, conducting surveillance and providing epidemiologic intelligence. *Capacities*, on the other hand, represent the resources—infrastructure, response mechanisms, knowledgeable and trained personnel that a public health system has to draw upon. Much of what public health preparedness organizations do in “peace time,” i.e. between events - planning, training, and acquiring equipment and supplies - is intended to build capacity for future emergencies [6].

This analysis is based on the experience of five EU countries (Croatia, Finland, Germany, Italy, and Spain) during the first phase of the pandemic, i.e. before the initiation of vaccination programs in December, 2020. They were chosen to illustrate different national organizational structures and responses to the pandemic, with consideration to the team’s familiarity with the countries’ characteristics (including language abilities), and the countries’ availability and willingness to participate. In the analysis, if feasible, we considered both problems encountered and adaptations made during and after the first wave.

This analysis draws on pandemic preparedness plans, reports, and other documents provided by the countries and other literature identified through conducted rapid literature reviews. In addition, we conducted interviews of individuals in each country knowledgeable about the areas of inquiry. This included discussions with ECDC National Focal Points (NFPs) for preparedness and response as well as other experts in the countries whom they suggest to be contacted. An interview guide was prepared based on a preliminary analysis of the literature, and Pawson and Tilley’s approach to conducting stakeholder interviews was employed [8]. Preliminary results were discussed in virtual meetings with ECDC’s NFPs for preparedness and response of the five countries participating in the project. Facts not specifically referenced are based on these interviews, but individuals are not identified for reasons of confidentiality.

### Limitations

This analysis is limited to the first phase of the pandemic (the year 2020, before the introduction of vaccination programs in the EU countries), and focuses only on two issues that a preliminary review identified as important in this stage of the pandemic in Europe: testing and surveillance. Coordination and communication within the public health emergency preparedness system, with other sectors such as education and civil protection, or at the international level are not addressed.

The analysis was limited to five countries, although they were chosen to illustrate different governmental systems that experienced the pandemic in diverse ways. Only EU member states that were willing to commit to participation are included. The number of individuals who were available to be interviewed was also limited. However, the literature was reviewed in the peer-review publications that in some cases went beyond the five countries, and the convergence in issues raised in the interviews and in the literature suggests that no major issues were not addressed.

The interviews were conducted primarily with governmental officials in health agencies at the national level. With few exceptions, we did not speak to regional or local officials or representatives of private-sector healthcare organizations; doctors, nurses, or other healthcare providers, or patients’ organizations. Although some of these perspectives were represented in the published literature and by the interviewees, other issues may have been missed.

## Results

### Laboratory analysis

In the ECDC logic model, the Laboratory analysis capability is defined as “The technical ability to identify (possibly novel) pathogens, monitor antimicrobial resistance, and to handle large numbers of samples submitted for diagnostic purposes.” Testing is fundamental to a number of other assessment capabilities and capacities. In 2020 the ECDC [9] identified five main objectives for testing: (1) controlling transmission of SARS-CoV-2, (2) reliably monitoring transmission rates and severity, (3) mitigating the impact of COVID-19 in healthcare and social care settings, (4) detecting clusters or outbreaks in specific settings, and (5) maintaining COVID-19 elimination status once achieved.

#### Testing capacity

On 23 January 2020, German scientists published a paper describing the development and validation of one of the first Polymerase Chain Reaction (PCR) tests to detect Severe acute respiratory syndrome coronavirus 2 (SARS-CoV-2) in patients. The test was based on the genetic sequence for the virus Chinese scientists had posted on 10 January 2020.

Germany was also able to increase testing capacity early because its laboratories have the expertise, accreditation, and equipment to conduct PCR assays and quickly deliver diagnoses. Clinical labs that normally deal with human samples required no additional licensing, but arrangements had to be made for research and veterinary laboratories, for example by removing the requirement that physician specialists sign the results. The speed and effectiveness of the deployment and evaluation effort also were enabled by national and European research networks established in response to international health crises in recent years, demonstrating the enormous response capacity that can be released through coordinated action of academic and public laboratories [10].

German public health budgets initially did not cover COVID-19 test costs, but on February 28, 2020 the federal government mandated that all insurance companies pay for tests for symptomatic people, which in turn incentivized private laboratories to scale up quickly. The policy was later updated to include asymptomatic individuals and to prioritize rapid tests for those in contact with high-risk people (e.g., a hospital or nursing home) [11].

When the first case in Finland was detected on January 29, 2020 [12], infrastructure and regulatory structures were in place so PCR testing was adapted very early. The assay was available first at national respiratory virus reference laboratory at the Finnish Institute for Health and Welfare (THL) and in the largest clinical microbiology laboratory in the Helsinki-Uusimaa hospital district, and shortly afterwards in the other four university hospital clinical microbiology laboratories. Prompt collaboration regarding regulatory matters with Finnish Medicines Agency (Fimea) facilitated the use of commercial assays when they became available. The clinical microbiology network was subsequently expanded, and eventually 30 laboratories were performing COVID-19 diagnosis.

Antigen tests were widely adopted in May 2020, in part because the test was developed and produced in Finland. During the first months of response, in Northern and Eastern Finland long distances caused delays in transporting samples to laboratories so these parts of the country quickly adopted local antigen testing when it became available. This choice reflected a trade-off between lower sensitivity but quicker results. In Southern Finland, on the other hand, PCR tests have been predominantly used and antigen tests have mainly been used by the private sector. Beyond that, the biggest challenges in scaling-up diagnostic testing were related to resources: recruitment of new personnel, procurement of laboratory equipment and facilities, sampling sites and test kits, and procurement of swabs and other laboratory consumables.

Italy drew on its existing laboratory network when it experienced large numbers of cases early in the pandemic. On January 22, 2020, a network of 31 laboratories was identified to diagnose suspected cases of SARS-CoV-2 infection according to the protocols indicated by World Health Organization (WHO). At the same time, the National Institute of Health (Istituto Superiore di Sanità - ISS) - (WHO National Influenza Centre - NIC/ISS) was identified as the national reference laboratory for confirmation and reporting to WHO of all cases of SARS-CoV-2 infection identified in Italy. Italy issued an alert at the European Union/European Economic Area (EU/EEA) level, documenting the evolving epidemic, making the data available publicly through international media interviews, online bulletins and in preprint scientific papers.

Initially, cases were tested both locally and at the national reference laboratory, but this double testing was discontinued once the accuracy of the regional labs’ accuracy was confirmed. There also were challenges related to the absence of testing kits and machines, new protocols, and subsequently to scalability issues. As of end of March 2020, food safety labs were also used to increase testing capacity. These labs are integrated at the national level and have a tradition of working with the Ministry of Health, which made collaboration easier.

Although public health guidelines are issued by the Ministry of Health, Italian regions are extremely diverse in terms of population characteristics and organisation of local health systems. All regions are required by law to provide basic public health functions including laboratory and surveillance, but some have a more hospital-driven approach, while others are more focused on the primary care. In addition, the virus spread hit some areas earlier than in others. Consequently, the guidelines implemented in the regions are adapted to the different local situations. For instance, following the guidance from public health authorities in the central government, Lombardy and other regions opted instead for a more conservative approach to testing. Through the end of March, 2020, Lombardy conducted half of the tests conducted in Veneto on a per capita basis, and had a much stronger focus only on symptomatic cases. Lombardy made less investments in proactive tracing, home care and monitoring, and protection of health care workers [13].

On the other hand, the Veneto Region started contact tracing and case finding early and expanded it further during the course of the epidemic [13]. To implement this strategy, the Veneto Region developed a comprehensive, population-based data linkage approach and a real-time data analysis that considers all information on confirmed cases, case contacts, isolations, clinical conditions and active surveillance. This required drastically scaling up testing capacity, targeting mildly symptomatic cases, focusing on home diagnosis and care, and tracing contacts as much as possible.

Drawing on this experience, Italian authorities published a strategy and planning guidance for prevention and response to COVID-19 for the autumn-winter season in October 2020. This document mentions a formal recognition in law and extensive enhancements to the COVID-19 National Reference Laboratory at the ISS and other labs to support more effective testing in the then anticipated second wave [14]. Similarly, the Italian plan for pandemic influenza, published in January 2021 highlights the importance of slowing transmission through extensive diagnostic testing and systematic contact tracing, which had not been recommended in the past. The plan also stresses the importance of developing specific, sensitive, and reproducible test for the rapid molecular diagnosis of the new viral pathogen and of sharing protocols for the development of the diagnostic assays [15].

Spain, which also has a decentralized health system with most public health authorities lodged with Autonomous Communes, experienced similar problems with testing. For instance, the case definition and testing strategy were subject to frequent changes in the early months of the pandemic. Spain initially had only one national reference laboratory performing SARS-CoV-2 testing. Early in February, other laboratories developed the ability to conduct PCR testing, but could only process small numbers of samples. Non-hospital labs were incorporated but their separate information technology (IT) systems led to problems in collaboration with public laboratories.

In reaction to this experience, the Spanish pandemic plan published in July 2020 includes a variety of elements to ensure sufficient diagnostic and control capacity to diagnose increased transmission in the population through the adequate functioning of the early warning system of rapid response and epidemiological surveillance. The plan recognizes the need for adequate laboratory capacity for surveillance and increased diagnostic demand so prioritizes the development of new diagnostic techniques. The need for coordination between different levels and bodies (health care, social health, public health, civil protection) for the adequate implementation of early diagnosis and control protocols is also recognized [17].

#### Contact tracing

The Italian Veneto Region started contact tracing and case finding early and expanded this further during the course of the epidemic [18]. To implement this strategy, the Region developed a comprehensive, population-based data linkage approach and a real-time data analysis that considers all information on confirmed cases, case contacts, isolations, clinical conditions and active surveillance. This required drastically scaling up testing capacity, targeting mildly symptomatic cases, focusing on home diagnosis and care, and tracing contacts as much as possible. Other regions of Italy, however, made less use of contact tracing.

In Germany, the health experts emphasized the fundamental importance of contact tracing and the need to maintain public health staff levels to keep caseloads low, so that contract tracers could keep up with the volume. For example, health authorities and scientists closely examined—and eventually broke—the chain of transmission among the first cluster of cases, which occurred at the end of January 2020 in Bavaria. The biggest need related to contact tracing was human resources at local public health facilities, many of which were understaffed. The German Federal Ministry of Health hired and the Robert Koch Institut (RKI) trained “containment scouts”— typically medical students—to support local authorities in tracing contacts. Citizen science projects were launched to complement the government’s efforts [11].

Spain, Croatia, and Finland [19] experienced similar challenges, and adopted different solutions including supplemental human resources, changing legislation, and information systems.

#### Laboratory analysis conclusions

The primary finding in this area is that being able to conduct testing at scale (a capability) was especially critical early in the pandemic in EU member states. The COVID-19 experience showed that the inability to scale up testing operations also created problems for public health operations (e.g. epidemiologic investigations, contact tracing), for situational awareness (e.g. when restrictions on movement could be introduced or lifted), and risk assessment and characterization. This required drastically scaling up testing capacity, targeting mildly symptomatic cases, focusing on home diagnosis and care, and tracing contacts as much as possible.

Countries’ ability to scale up testing depended in part on a variety of existing capacities: the ability of university, hospital, and commercial laboratories to develop a test for a new pathogen, and of regulatory structures to approve it; the availability of swabs, reagents, and other supplies and the flexibility to obtain such in a timely manner; having or rapidly training staff to conduct testing at scale; and the existence of an electronic reporting network that is interoperable at different organisational levels and includes public and private entities. It also depended on the flexibility and resilience of the public health system, especially regulatory agencies, to approve a new test; to identify additional sources of lab capacity, such as harnessing public health, university, hospital, commercial or veterinary lab capacity to be used for COVID-19 testing; and to develop guidance regarding when and which tests should be used.

The German and Finnish experience illustrates the importance of pre-existing capacity in industry, academia, or public health laboratories to develop and deploy a test for a novel pathogen as well as the flexibility to quickly adapt to the circumstances.

Several government agencies designed rapid regulatory processes for emerging infectious diseases such as an emergency use authorization (EUA) programs to expeditiously authorize certain products for emergency use.

### Surveillance and epidemiological monitoring

In their analysis of lessons learnt from easing COVID-19 restrictions, Han, Tan, et al., [21] identify the importance of knowing the population’s infection status and having indicators to monitor the epidemiological situation, both of which depend on testing, especially after the recognition of the asymptomatic transmission of the virus [22]. This means real-time data of high quality are essential to calculate the reproduction number (R) and to track the disease spread in order to enable differential, targeted response [21].

Germany’s focus on collecting and analyzing data and communicating the results to the public contributed to high levels of trust in the government throughout the first half of 2020 - the very first phase of the pandemic. This began in January, 2020, well before the WHO’s declaration of a Public Health Emergency of International Concern (PHEIC). The Government regularly cited RKI surveillance data reports and used epidemiological concepts such as the reproduction rate as a driving factor behind decisions related to social distancing measures. In May 2020, the country moved forward with relaxing its physical distancing guidelines based on data collected on case counts and growth. The German government has focused on three indicators—infection rate, disease severity, and health system capacity—to monitor the pandemic and to introduce respective response measures. Setting clear expectations and providing transparency to the public on the criteria for government decision-making (e.g. about reopening of the country) was considered as a key factor in gaining public trust [11].

Germany’s federal system led to varied approaches and guidance applied at local level related to social distancing. While this allows for tailored strategies, it has also limited widespread implementation of a standard testing strategy or nationwide containment measures even in the face of rising case counts. Coordination of efforts was difficult, especially given fixed responsibilities represented in the national law on infectious diseases. In response to COVID-19, there have been changes in some legislative acts to give the federal government more power in an emergency situation [11].

In Italy, data availability was a problem, especially at the onset of the pandemic. Pisano, Sadun, and Zanini [13, 18] have suggested that that the widespread diffusion of the virus in the early months of 2020 may have been facilitated by the lack of epidemiological capabilities and the inability to systematically record anomalous infection peaks in some hospitals. Ideally, data documenting the spread and effects of the virus should be as standardized as possible across regions and follow the progression of the virus and its containment at both a macro (state) and micro (hospital) level. The reported differences in mortality rates between Italy and other countries and among Italian regions may be driven (at least in part) by different testing approaches [13]. This might have complicated the management of the pandemic, because in absence of comparable data it is hard understand what measures work and where more resources need to be allocated.

To address these concerns, the Italian COVID-19 response plan issued in October, 2020 describes the development and implementation of an Integrated Surveillance System. A web platform incorporated information from, and facilitated data sharing with, the Department of Civil Protection (which prepares daily aggregated COVID-19 case counts), an existing influenza surveillance system (InfluNet), virological surveillance, and other systems. This platform is designed to manage the national rapid alert network, along the lines of the European Commission’s Early Warning Response System (EWRS), in which national and regional entities can communicate promptly with a guarantee that sensitive data is adequately protected. The system includes individual-level data on positive cases (following adequate procedures to guarantee confidentiality and data protection), which allows for analysis by region, origin and setting, and for vulnerable populations. The plan also seeks to standardize methods for contact tracing in general and particularly in school outbreaks, which should help to harmonize the surveillance data that come from these systems [14]. The system was developed by the ISS in collaboration with the Italian Ministry of Health and the regional and local health authorities [23].,

Similarly, the Italian influenza pandemic plan published in January 2021 [15] highlights the importance of sharing data collected through testing and other activities, carrying out a preparedness activity by developing “framework” operational protocols and tools for data collection in the inter-pandemic phase. For instance, in order to put in place adequate measures with respect to different levels of risk during the transition from the first phase of the pandemic to the so-called phase 2, indicators were established for comprehensive monitoring activities [24]. Process indicators to monitor capacities and outcome indicators related to transmission and maintenance of health services were also included.

The Spanish Ministry of Health and the Autonomous Communes developed a protocol for the management of COVID-19 cases and surveillance guidelines on January 22, 2020 [16]. Samples, as well as clinical and epidemiological information, were collected at a regional level and data were entered electronically in the Spanish Surveillance System (Sistema de Vigilancia en España - SiViES). During the first wave of the pandemic, however, the Autonomous Communes had different data systems that were incompatible with each other. It was not possible at this time to use SiViES to compile data for the whole country, so a simplified protocol was developed to share aggregate data (with 5–7 variables) on a daily basis. Consequently, there were delays and inconsistencies in surveillance data. A national IT system was developed later in 2020, and epidemiologists used seroprevalence surveys to retrospectively reflect epidemiological situation in the early months of the pandemic.

In order to strengthen early detection, investigation and transmission control, a robust legal framework was established in June 2020 for prevention, containment, and coordination measures to deal with the public health crisis caused by COVID-19 [25]. In particular, local and regional public health authorities were deemed responsible for COVID-19 outbreak detection, investigation, and control, early identification and quarantine of contacts, as well as implementation of control measures in settings where outbreaks were occurring. Based on the national framework, all regions were to notify at national level every identified COVID-19 outbreak by email using a specific template [26]. All notifications were compiled in a database and analysed daily at the Coordinating Centre for Health Alerts and Emergencies (CCAES) at the Ministry of Health (MOH). During the pandemic, the MOH also took responsibility for the National Epidemiological Surveillance Network (Red Nacional de Vigilancia Epidemiológica), which is normally under the auspices of the National Centre of Epidemiology (Centro Nacional de Epidemiología). Every day, an internal report was distributed within the National Surveillance and Alert Network describing the demographic and epidemiological characteristics of the outbreaks. Twice a week, a summary of the outbreak situation report was made publicly available on the MOH web site and was presented in the technical press conferences hosted at the MOH [26].

In Croatia, testing, contact tracing, and surveillance are organized at two levels. Each of 21 counties has its own public health institute and lab, and these were charged with testing and contact tracing. The National Institute for Public Health issued guidelines for contact tracing, surveillance and laboratory testing and provided national coordination. A platform was specifically developed during the pandemic to collect information and was located at the National Insurance Fund, which was responsible for financing testing and diagnostics. Hospitals used this platform as well for COVID-19 testing, sending information to the Croatian Institute of Public Health (CIPH) for contact tracing.

In Finland, THL is responsible for providing guidance, but the local level has the power and responsibility to implement control measures, such as quarantine. Infectious disease specialists in hospitals and municipal services are responsible for reporting to the national level via the national registry for infectious diseases. There are two registers – lab and medical – in which COVID-19 cases had to be added. Some data were added based on surveys (data coming from the labs). Hospital registers existed, but were not used online. Initially, data on COVID-19 laboratory tests were collected directly from the laboratories and hospitals conducting the tests. As the pandemic evolved, however, the number of tests and test results were reported through the National Infectious Disease Register maintained by THL [20].

In the early spring of 2020, the experts started to put data in the system as patients entered hospital, not when they were discharged or died. Starting in March 2020 reporting from hospitals on cases and mortality was daily; from June 2020 onwards the reports were three times per week. In the initial stages of the pandemic, it was not clear how many of the cases were serious, e.g. required intensive care, and it was a challenge to develop a registry to collect these data. The existing system was not designed for on-line reporting on a daily basis, so changes were needed to add specifications and document templates for daily reporting.

The numbers of COVID-19 patients treated in hospitals and intensive care units (ICUs) have been collected through the hospital districts. The data on ICU utilization was collected by the Intensive Care Coordination Office of the Kuopio University Hospital for the Finnish Intensive Care Consortium. The hospital districts also report data on COVID-19 related infections and deaths in their region. THL and hospital districts report data on a different basis – THL reports data based on the patient’s residence, while hospital districts report the test results that have been done in their area. The reporting system has generally functioned well, but in early days there were misconceptions about reporting responsibilities in some areas, which resulted in problems when reporting deaths in elderly care institutions (which was later corrected).

In early April 2020, THL started to investigate the spread of coronavirus in the population using antibody assays based on random sampling and in collaboration with university hospitals in order to examine to what extent the epidemic has spread in different age groups and regions [27]. THL regularly published results on its website [28], however, seroprevalence remained low and did not prove to be very informative. Indeed, it has been subsequently refocused on more targeted groups – vulnerable groups who might not being seeking testing options.

#### Surveillance conclusions

Providing national surveillance data during the first months of the COVID-19 pandemic was challenging in two respects: modifying existing infectious disease reporting systems to add a new “notifiable” disease and developing or modifying ad hoc surveillance systems (e.g. hospital capacity, syndromic surveillance).

These challenges were due in part to the hierarchical structure of national public health systems, or different governance organisation and reporting lines in the public health and health care institutions. Although the specific organizational structure and responsibilities vary, these systems typically have a national public health lab and regional authorities (e.g. German Länder), with local (e.g. municipal) administration by public health agencies and hospitals. Case reports are generated by primary care physicians and hospital providers, which are typically not formally part of the public health system. As a result, making the necessary changes to the infectious disease surveillance systems required extensive communication and coordination among many entities at different levels of the hierarchy and in multiple geographic areas. In at least one country (Germany), legislative changes were needed to change surveillance systems. As with laboratory testing, flexibility and adaptability were key. Although there are benefits to a decentralized approach, coordination of efforts at national level was difficult, especially given fixed responsibilities represented in the national law on infectious diseases on timely reporting.

Similar to the conclusions on testing, countries’ ability to adapt existing and develop new surveillance systems is a capability that must be executed under pressure. Their success depended in part on the prior existence of disease notification requirements and reporting protocols, electronic reporting systems, and epidemiologists with the appropriate analytical skills. But as with laboratory capacity, flexibility and adaptability was critical. National, regional, and local public health systems had to change reporting requirements and develop new ways to share and analyse data.

### Risk characterization

With respect to communicable diseases, ECDC logic model defines Risk characterization as “identifying the (possibly novel) pathogen and its epidemiologic characteristics such as modes of transmission, risk of infection, virulence (e.g. case-fatality rate), intergenerational time, control strategies, and so on.” The experience in the EU countries, in focus of this project, confirmed the importance of this capability. In Vo’ (Veneto, Italy), for example, early investigations were carried out during two separate surveys on the majority of the population (85.9% and 71.5%, respectively) throughout nasopharyngeal swab identifying a large proportion of asymptomatic individuals among the infected people [29]. This was a crucial finding to explain the spreading of the SARS-CoV-2 infection, differently than other coronaviruses as SARS-CoV-1 and MERS.

The Robert Koch Institute’s ability to characterize and quickly conduct epidemiological analyses and contributed to international efforts to characterize COVID-19 risks helped to inform disease control policies and provided necessary information for public risk communication [11]. RKI publishes risk assessments, strategy documents, response plans, daily surveillance reports on COVID-19, and technical guidelines, communicating this information via national and international public health authorities. This steady flow of information has helped the government—as well as local and intermediate public health authorities, health professionals, and the public—make critical decisions during the outbreak [11].

To address concerns identified in the initial months of the pandemic, the Italian plan for the prevention and response to COVID-19 in the autumn-winter season issued in October, 2020 [14], describes a monitoring system for the quantitative classification of the risk and resilience of regional public health and healthcare systems that has been implemented by the ISS and coordinated by the Ministry of Health. The system includes regular consultation mechanisms with technical contacts within regional health systems and with a national coordination committee (“Cabina di Regia”). In order to monitor the quality and completeness of the information reported by the regions and autonomous provinces and to provide them with a tool to check their data quality, automatic reports are produced weekly and sent to each region and autonomous province reporting missing/inconsistent data for each indicator so they can be resolved. The Italian influenza pandemic plan published in January, 2021 [15] established the Italian influenza pandemic preparedness network made up of public health representatives of the regions and autonomous provinces as well as representatives of relevant institutions. This reference network provides for a dynamic approach to preparedness that can interface with the well-established and equally expected epidemiological and virological networks for indicator-based and event-based surveillance (for example the international InfluNet network and the Italian epidemic intelligence network). It is expected that the Italian influenza pandemic preparedness network and all the institutions involved will be able to benefit from *ad hoc studies* on the potential impact of the pandemic influenza pathogen at pandemic risk on the Italian population and health services through the establishment and activation of a multidisciplinary network of experts called DISPATCH who focus on epidemic intelligence, pandemic scenarios, risk assessment. The new influenza plan also outlines the importance of research and development activity of the genetic sequencing capacity of the National Influenza Centre of the ISS on strains of new respiratory viruses.

In June, 2021 the Italian Ministry of Heath established the Epidemic Intelligence Network, an event-based surveillance system, to coordinate all activities aimed at the early identification of risks in public health, their investigation, validation and evaluation. Although part of the pandemic influenza plan, it is anticipated that the Network will be used to monitor other emerging pathogens. The Network also can be activated by the Ministry of Health to monitor the evolution of international pandemic alerts by creating situation awareness reports suited to national information needs [30].

## Discussion

The European experience in the first wave of COVID-19 demonstrates that a country’s ability to conduct testing at scale was critical, especially early in the pandemic. The inability to quickly scale up testing operations also created problems for public health operations such as contact tracing, for situational awareness, and risk assessment and characterization. COVID-19 also required public health emergency preparedness systems to develop new strategies, approaches, and policies under pressure and to review and revise them as the pandemic evolved.

Countries’ ability to scale up testing depended on a variety of existing capacities: the ability of public and private laboratories to develop a test for a new pathogen, and of regulatory structures to approve it; the availability of swabs, reagents, and other supplies; having or rapidly training staff to conduct testing at scale; and the existence of an electronic reporting network that includes public and private entities. It also depended on the flexibility and resilience of the public health system, especially regulatory agencies, to approve a new test; to identify additional sources of lab capacity, such as harnessing public health, university, hospital, commercial or veterinary lab capacity to be used for COVID-19 testing; and to develop guidance regarding when and which tests should be used.

Although our analysis was limited to five countries and a particular period of time, the findings are consistent with other analyses of the COVID-19 experience in Europe and elsewhere. For example, a report to the European Parliament in 2021 identified difficulties that were encountered in accessing comparable data and suggested that the Union provide support to Member States to ensure the collection and sharing of data in times of health crisis [31]. A more recent summary identified the need for improved collection and analysis of data and evidence as one of four major lessons learned from the pandemic [32]. As noted above, in their analysis of lessons learnt from easing COVID-19 restrictions in high-income countries in the Asia Pacific region and Europe, Han, Tan, and others identify the importance of knowing the population’s infection status and having indicators to monitor the epidemiological situation, both of which depend on testing. These data are needed to track the disease spread in real-time in order to enable differential, targeted response [21]. The Lancet Commission on lessons for the future from the COVID-19 pandemic similarly concludes that public health systems require strong surveillance and reporting systems [33]. Similarly, Rajan, Cylus and McKee discuss the complexities of implementing a testing program, and the implications for a successful find, test, trace, isolate, and support (FTTIS) strategy [34].

The COVID-19 experience also reminds us that public health and health care systems operate at the national, regional, and local level with respect to testing, contact tracing, and surveillance, and involve both government agencies as well as private organizations. Consequently, communication among multiple public and private entities at all levels and to coordinate their testing and surveillance activities also is critical. The ability to coordinate efforts between levels (national, regional local, organisational) during a public health emergency depends on the existence of communication channels (e.g. for communicating testing policies) and data systems (e.g. for sharing surveillance data). Countries had to develop or modify ad hoc solutions to these problems in the early days of the pandemic. Having pandemic preparedness plans in place before an emergency or systems that could be modified as necessary helped, and thus is an important preparedness capacity.

These issues go beyond testing and surveillance. For example, the broader report to which this analysis contributed also addressed health sector coordination and emergency risk communication. It found that to a degree not contemplated in existing preparedness measures, COVID-19 required EU Member States’ PHEP systems structures to develop new strategies, approaches, and policies under pressure and to review and revise them as the pandemic evolved. The report also found that existing preparedness measures generally do not reflect (a) countries’ internal hierarchical structure of public health, healthcare, and other entities that influenced emergency responses; (b) the required coordination among the different sections of the healthcare system, particularly hospital and community-based level or (c) existing preparedness measures generally do not represent the challenges of scaling up a country’s pandemic response [4].

More generally, Bollyky and colleagues, among others, demonstrated the importance of government and interpersonal trust in determining COVID-19 outcomes and called for improved risk communication and community engagement strategies to boost the confidence that individuals have in public health guidance [35]. The risk communication component of our project found similar results [3, 4]. Trust also depends, we believe, on governmental agencies having and sharing reliable data on the epidemiologic situation, risks, and other factors. In this respect, improvements in testing, surveillance, and epidemic intelligence suggested by this analysis represent an important contribution to creating the conditions for good governance and trust required for an effective pandemic response.

## Conclusions

COVID-19 pandemic provides a unique opportunity to learn the challenges encountered by public health emergency preparedness systems, both in terms of problems encountered and adaptations during and after the first wave, as well as successful responses to them. With regard to testing and surveillance, three capabilities that were essential to the COVID-19 response in the first phase, and presumably in other public health emergencies: the ability to scale-up testing, contact tracing, surveillance efforts; flexibility to develop new strategies, approaches, and policies under pressure and to review and revise them as the pandemic evolved; and the ability to coordinate and communicate in complex public health systems that operate at the national, regional, and local level with respect and involve multiple government agencies as well as private organizations.

## Data Availability

Data sharing is not applicable to this article as no datasets were generated or analysed during the current study.

## Abbreviations

ARI: Acute respiratory illness
CCAES: Coordinating Centre for Health Alerts and Emergencies
COVID-19: Coronavirus disease 2019
CIPH: Croatian Institute of Public Health
EWRS: Early Warning Response System
EU: European Union
EU/EEA: European Economic Area
ECDC: European Center for Disease Prevention and Control
EUA: Emergency use authorization
Fimea: Finnish Medicines Agency
ICUs: Intensive care units
ISS: Istituto Superiore di Sanità
IT: Information technology
MOH: Ministry of Health
NFPs: National Focal Points
PCR: Polymerase Chain Reaction
PHEIC: Public Health Emergency of International Concern
RKI: Robert Koch Institut
THL: Finnish Institute for Health and Welfare
WHO: World Health Organization
